# Improving HPV vaccine acceptance through peer-to-peer education among adolescent girls in the urban poor settings of Kisenyi, Kampala, Uganda

**DOI:** 10.1371/journal.pgph.0004007

**Published:** 2024-12-05

**Authors:** Doreen Tuhebwe, Christine Adyedo, Emmanuel Ahumuza, Steven Ssendagire, Rhoda K. Wanyenze

**Affiliations:** 1 Makerere University School of Public Health, Kampala, Uganda; 2 Mukono District Local Government, Mukono, Uganda; 3 Ministry of Health, UNEPI, Kampala, Uganda; NYU Grossman School of Medicine: New York University School of Medicine, UNITED STATES OF AMERICA

## Abstract

In Uganda, cervical cancer due to Human Papilloma Virus (HPV) is the most prevalent cancer among women. In 2015, the HPV vaccine was introduced into Uganda’s EPI program, targeting young girls in-and out-of-school. However, HPV vaccine uptake remains low at 44% for the second dose with disparities in vulnerable populations in urban poor settings. We piloted a peer-to-peer education approach in the urban slums of Kisenyi in Kampala, Uganda to address vaccine hesitancy among adolescent girls aged 9-13years. In 2019/2020, a total of 18 girls between the ages of 10–15 years old, who had previously received two doses of the HPV vaccine were trained as Adolescent Peer Educators (APEs), co-created an HPV vaccine health education message, and conveyed it to unvaccinated peers, with the intention of increasing vaccine uptake. The APEs attended weekly mentorship meetings with the intervention team to document their experiences and be supported to link interested peers to the nearest public health facility for vaccination. Over a 12-week period, the APEs identified 192 unvaccinated peers aged 10-13years, 177/192 were provided with the health education messages, 145/177 expressed willingness to receive the first dose of the vaccine and 88/145 (60.7%) received it. Through repeat socialization, positive influence and friendship, the APEs were able to communicate the benefits of the HPV vaccine, manage fears like anticipation of injection pain and connect interested peers to the community health workers (CHWs) for health facility linkage. A common barrier experienced by APES was the fact that caretakers made the final decision even after their daughters expressed interest requiring CHW intervention with caretakers. Peer-to-peer education and linkage to vaccination is a feasible approach that could increase uptake of HPV vaccine among adolescents. We recommend larger scale effectiveness studies to refine the model and include a comparison group to identify the optimal intervention components.

## Introduction

Globally, cervical cancer is the fourth most common cancer in women with over 90% of the burden in developing countries, and Sub-Saharan Africa having the highest rates in the world [1]. Of those affected 85% are young, under educated women who live in the world’s poorest countries, reflecting global inequities [2]. In Uganda, cervical cancer is the most frequently occurring cancer among women and has shown an increase of 1.8% per annum for the last twenty years [3]. The 90-70-90 WHO 2030 targets to eliminate cervical cancer aim to have 90% of girls fully vaccinated with the HPV vaccine by the age of 15 [2]. It is estimated that the human papillomavirus (HPV) vaccine programme would reduce deaths from cervical cancer by two-thirds if uptake reached 80% [4]. Initially, two vaccines to prevent human papillomavirus (HPV) infection were approved for use in over 120 countries targeting adolescents aged 9 to 14 years and currently, the World Health Organization (WHO) has licensed six HPV vaccines [5]. However, only one in eight girls has received the HPV vaccine globally [6].

In Uganda the HPV vaccine program targets in- and out-of-school girls aged 9–13 years free of charge [7]. The annualized coverage of HPV1 is 85% and HPV2 at 41% in 2019 and stagnating at 75% and 44% respectively in 2022 [8, 9]. Uganda’s HPV vaccine coverage is not homogenous with some regions in rural and peri-urban areas having lower uptake [10, 11] and further lower among disadvantaged groups that live in poverty, with lower education and health literacy, with generally less access to health services—characteristics that are typical in urban poor setting like Kisenyi slum in Kampala, Uganda where we conducted this study. In 2018, HPV2 (second dose of HPV vaccine) coverage was as low as 47% in Kisenyi Parish, yet the vaccine is provided at the nearby public health facility and through school and community outreaches in Kisenyi. This low uptake has been attributed to vaccine hesitancy where people including parents, teachers, caretakers and adolescent girls have conflicting or opposing views towards vaccination (intentions and willingness), lack of knowledge about the vaccine and its benefits, socio-economic inequities, frequent mobility and migration which affects continuity of and linkage to care and health system access challenges such as long waiting times and culturally nonadaptive service delivery [10, 12–16].

Gaps in utilization of immunization services among urban residents have also previously been attributed to access challenges [17], despite the availability of service delivery points, especially for the HPV vaccine. This is sometimes compounded by the fact that vaccination has traditionally targeted children and not healthy young people, and the HPV vaccine deviates from this expected norm and may thus require different service delivery models and platforms to reach adolescents [18]. Programs must also address problems such as lack of trust in the people and institutions delivering vaccination programs and the spread of rumors and myths around vaccines among other challenges [18, 19]. Low HPV vaccine uptake compromises achieving the benefits of the vaccine and the elimination of cervical cancer, especially among vulnerable populations such as women living with HIV and in places such as slums where young girls have early sexual debut before age 15 [20, 21].

Few studies have designed and tested interventions to address vaccine hesitancy [22] and this has inspired calls for social science perspectives in understanding and addressing the problem [23], especially in low- and middle-income countries (LMICs) like Uganda, where the HPV vaccine is relatively new. WHO has recommended the use of the behavioural and social drivers (BeSD) of vaccination framework to understand reasons for low vaccination coverage and to inform targeted interventions [24]. The BeSD framework scholars identified vaccine champions and advocates as social process interventions that work as parents and friends have been shown to influence vaccination uptake decisions of young people [25, 26].

Drawing on Bandura’s Social Learning Theory [27], adolescents can look at their peers as major sources of social and emotional support pointing to the potential of adolescent peer educators (APEs) being a source of knowledge about the HPV vaccine when empowered with the right content, community access and they themselves have experienced vaccination—thus can recommend it and aid the decision-making process through referral [28–30]. Peer-to-peer education approaches have been effective in the control of HIV/AIDS, the fight against gender based violence and considered a health promotion strategy in adolescents since youth social groups and peers can influence or encourage each other to get involved and participate in new activities including vaccination and cancer screening uptake [31–34].

We applied the concept of peer-to-peer education to empower vaccinated adolescent girls to become change agents and influence fellow girls in Kisenyi slum to take up the vaccine as part of a social behavioural intervention and documented the processes and outcomes to inform future interventions to address vaccination hesitancy and improve HPV vaccine acceptance in low- and middle-income countries.

## Methods

### Study setting

This study was conducted in Kisenyi Slum, in Kampala city. Kampala city has the highest urban population growth rate in Uganda [35] and Kisenyi slum is the largest slum in the center of Kampala, with typical urban poor characteristics of; 1) having informal settlements, 2) having clusters of dilapidated housing, 3) a large population size and 4) rapid expanding population [36] with an administrative composition as described elsewhere [37]. Kisenyi II Parish which is more residential was purposively selected for this study to give a representation of a typical Ugandan adolescent girl that resides in Kisenyi. Eight zones (villages) out of 10 in Kisenyi II were included in this study. Kisenyi Health Center IV is the only government funded public health facility in the area and provides routine vaccination (including HPV vaccine). Outreaches are held in schools in partnership with the Ministry of Education and Sports and in the community to reach girls out-of-school. In the community, the village health teams (VHT)- a level of community health workers in Uganda, visit caretakers of the targeted girls (aged 10-13years) to mobilize them to take up the vaccine.

### Adolescent peer educator selection

The sample for this study was girls aged 10-15years living in Kisenyi slum for the past one year and had received two doses of the HPV vaccine according to the Uganda Ministry of Health policy [38]. The intervention team worked with the eight VHTs from the eight zones of Kisenyi to conduct house-to-house visits and map the eligible girls. The house-to-house mapping followed by the recruitment exercise lasted four days: 3^rd^ - 6^th^ October 2019. The VHTs identified 62 adolescent girls in Kisenyi II that had received at least one HPV vaccine dose. Of these, 32/62 had received two doses of the HPV vaccine (completed vaccination) hence eligible to be an adolescent peer educator. The VHTs explained to them the objectives of the study and sought assent and caretaker consent for their recruitment as peer educators and 28/32 consented. Those that consented participated in needs finding (problem diagnosis) discussions with the intervention team from Makerere University School of Public Health (MakSPH) composed of two female scientists (DT and CA) with Master of Public Health training, were born and raised in Uganda and knew the local language. Three discussion groups (1.5 hours each) attended by 8–10 girls were held to explore the motivation to vaccinate and participate as peer educators. The discussions also identified the needs and barriers to HPV vaccination based on the discussion guide in S1 Text. After the discussion, 18 girls expressed interest in becoming adolescent peer educators (APEs) and the intervention team engaged with the VHTs to ensure representation of the APEs across the eight zones of Kisenyi II as well as the community cultural composition and trust by the VHTs. The mean age of the 18 APEs was 11.9(±1.5) years, they were all in school and 12/18 had received all their HPV vaccine doses from school.

### Cocreation of the peer-to-peer education intervention, piloting and evaluation

Following the selection of the APEs, the intervention team collaborated with the APEs to co-create a structured health education message (the message) using the human centered design (HCD) approach. The HCD approach as applied in immunization programs is a cyclic process that follows four stages; *Diagnose* (to understand the challenge), *Design* (turning insights into solutions), *Implement* (planning and acting and monitoring progress regularly and *Evaluate* (checking that the intervention is achieving the set goals) [39].

#### Diagnosis and design

We drew upon the needs finding discussion findings and the constructs of the Health Belief Model [40] as data for the diagnosis phase. The intervention team first presented the discussion findings and co-created with the APEs. The findings showed that the main enablers of vaccination were receiving parent’s advice, receiving friend’s advice and the vaccine being available or being brought to school. Barriers to HPV vaccination included misinformation that the vaccine causes infertility and lack of information about the benefits of the vaccine.

After sharing the findings from the needs finding discussion, the HPV health education message co-creation process followed. This was informed by the constructs of the Health Belief Model [40] namely; perceived susceptibility, perceived severity, perceived benefits, perceived barriers, cues to action and self-efficacy and the wording of the messaging was informed by the findings from the needs finding discussion. The co-creation process aimed at developing a message that communicates the risks of HPV, the benefits of the HPV vaccine, that the APE has previously received the vaccine, the vaccine is safe and how the vaccine can be accessed. After co-creation of the message, the APEs were trained over two days by the intervention team on how to identify girls aged 10-13years, confidently inquire about their peers’ HPV vaccination status and number of doses received and deliver a structured health education message to unvaccinated peers. Through engagements, the APEs and intervention team set a target for each girl to influence at least 10 unvaccinated peers for HPV vaccination over a 12-weeks period. See attached S2 Text for details of the APE training process and final health education message.

#### Implementation and evaluation

Over a 12weeks period (November 2019 to February 2020) the APEs attended a one-hour mentorship session on the weekend (Saturday) with the intervention team to share stories and lessons about their weekday engagements with girls in the community. During the mentorship session, the intervention team asked about 1) how many girls the APE approached, 2) how many unvaccinated girls were identified, 3) how many girls the APE shared the health education messages with, 4) how the conversation went, 5) whether the unvaccinated girl was willing to be vaccinated, 7) number of girls permitted by their caretakers to receive the HPV vaccine, 8) number of interested and willing unvaccinated peers linked to the VHT, 9) reasons why some of the unvaccinated peers refused to be vaccinated, and 10) the challenges faced during the peer-to-peer education. Over the 12-weeks, each mentorship meeting was attended by an average of 11 APEs ranging from 5–17 in a meeting.

The discussions held during the mentorship sessions were audio recorded with the assent of the APEs and later transcribed. Bandura’s Social Learning Theory principles [27] which emphasize learning by observing other people’s behaviour and experiences (in a social setting) were applied by the intervention team to structure the mentorship sessions and storytelling, facilitate group learning and encourage peer feedback when challenges were discussed. Group learning was supplemented with additional training by the intervention team on how APEs could leverage their social connections with fellow girls in the community to effectively advocate for HPV vaccine uptake and to strengthen the APEs’ communication and confidence skills. Based on the stories shared by the APEs, the intervention team and APEs reviewed the health education messages to correct any misinformation, and the message was standardized in week four to highlight that the HPV vaccine injection is not *very* painful.

Unvaccinated peers who were interested and willing to be vaccinated were linked to the nearby health facility through the VHT. The urban authority and local leaders in Kisenyi Slum and Kisenyi HCIV were engaged to ensure availability of the HPV vaccine throughout the study period. On every Wednesday of the 12-weeks period, Kisenyi HC IV provided HPV vaccination to unvaccinated girls that accepted to receive the vaccine. On the day of vaccination, the unvaccinated peers either came with their caretaker to the health center or came in company of the community health worker or the APE(s). The vaccines were free of charge and the newly vaccinated peers were provided with a purple vaccine card that indicated the date of first dose and date of the next dose. The intervention team also collected socio-demographic information from the newly vaccinated peers using an excel worksheet.

After the 12-weeks, the newly vaccinated girls (dose 1 or completed two doses) where invited to participate in focus group discussions (FGDs) upon consent of their caretakers and assent of the girls. The intervention team asked each of the newly vaccinated girls the name of the APE that referred them for HPV vaccination, how many doses they had received, the date for their next dose (if applicable), the reasons why they accepted to be vaccinated and their perceptions towards the benefits of the peer-to-peers education approach and APEs based of the guide shown in S3 Text. A total of six FGDs were conducted, each lasting one hour.

### Data management

The FGD guide(s) were translated into the local language prior to data collection. The Investigators led the discussions and asked open-ended questions followed by probes on predetermined themes. Flexibility was employed to explore emerging themes. All audio recordings were done with assent and consent of the participants and their caretakers respectively. The interview venue was selected in consultation with the VHTs and the FGDs, peer education training and weekly mentorship meetings were conducted in Luganda, the most spoken local language in Kisenyi. The discussions from the mentorship meetings and the FGDs with the newly vaccinated peers were transcribed verbatim and concurrently translated from Luganda without altering the meaning.

### Data analysis

Data for this study included audio recordings and field notes of the discussions held during the APE mentorship sessions with the intervention team, quantitative data from the weekly vaccination sessions held at the Kisenyi HC IV and the transcripts of FGDs with the newly vaccinated peers. The transcripts were analyzed using an excel matrix. The qualitative analysis was done in two stages; first, the manifest content analysis and then the latent content analysis [41] looking out for theme saturation. The transcripts were read, and codes were derived by highlighting emerging issues based on our understanding of the data. Codes were then sorted into categories based on their linkages. The categories were grouped together to inform meaningful overarching emerging themes that linked with the specific study objectives 1) identify the most significant stories of success or failure in the peer-to-peer education approach for increasing HPV vaccination acceptability, 2) explore the reasons contributing to the success or failures of the peer-to-peer education approach and 3) identify best practices by the APEs that optimize the peer-to-peer education approach. Quotes that represent the most significant stories on the role of peer educators in HPV vaccine acceptance were presented to support results under the respective categories. Descriptive analysis of the socio-demographic characteristics of the newly vaccinated peers was conducted.

Throughout the study, the team adhered to the RATS guidelines on qualitative research in terms of 1) the Relevance of study design in answering the research question; 2) Appropriateness of qualitative method such as use of FGDs and KIIs; 3) Transparency of procedures in sampling respondents and 4) Soundness of interpretive approach as applied to the data analysis [42]. We also completed the COREQ checklist in S1 Checklist. The Lead Researchers TD, AC, AE, and SS conducted the coding, data analysis and synthesis. The quantitative information about the newly vaccinated peers was analyzed descriptively and presented in frequencies and/or proportions. Where appropriate, quantitative results from the newly vaccinated peers, and qualitative results from the APE mentorship sessions or the FGDs were presented together for triangulation and to enhance contextualization of the results [37, 43]. A S1 Table has been included to share the diversity of quotes from the qualitative data analysis.

### Ethical considerations

This study was approved by the Makerere University School of Public Health Higher Degrees Research and Ethics Committee (Ref 717) and the Uganda National Council of Science and Technology (SS 5155). Permission was sought from Kampala Capital City Authority (KCCA). The Peer Educators participated voluntarily. Caretakers of the girls provided written informed consent and the VHTs led the identification of potential APEs in their community. All the girls provided written assent to participate in the study and were supported by their community VHTs in all the study processes but the VHTs did not attend any of the focus group discussions to ensure confidentiality. Any vaccine or vaccination myths and misinformation identified was corrected during the discussions with the APEs and the newly vaccinated peers. On the day of the mentorship meeting, each APE received a transport refund of $1.5USD and the VHT received $5 USD. A community dissemination meeting was held with the APEs, VHTs and Kisenyi Health Center IV representatives to discuss findings from the study.

## Results

This section describes the uptake of the HPV vaccine among identified unvaccinated peers, presents examples of significant stories of successes and failures in the APE approach, reasons for the success and reasons contributing to the failure of the APE approach.

### Vaccine uptake by identified unvaccinated peers in the community

By end of the 12-week period, 192 unvaccinated peers aged 10-13years were identified by the APEs, 177/192 were provided with the health education messages and 15/192 refused to listen to the APEs. Among those that received the health education messages, 145/177 expressed willingness to receive the first dose of the HPV vaccine and 88/145 (60.7%) received their first dose of the vaccine and got scheduled for the second dose. In a 2x2 table (Table 1), considering that 15 peers that did not receive the health message and were not vaccinated, and 88/177 who received the message got vaccinated, the data shows that there is a significant association between receiving the health message from the APE and HPV vaccine uptake (Fisher exact test P = 0.0001) without adjusting for confounders at significance level P <0.05.

**Table 1:** A 2X2 table visualizing the relationship between exposure to the health message and outcome of receiving the HPV vaccine.

	Outcome		
Exposure	Received HPV Vaccine	Did not receive vaccine	Marginal Row Total
Received health message	88	89	177
Did not receive health message	0	15	15
**Marginal Column Total**	88	104	192 (Grand Total)

The mean age of the 88 newly vaccinated girls was 11.9 years and majority (80/88; 90.1%) were in school. The most common reason for not vaccinating previously was “they did not know about the vaccine” (40/88; 45.5%). All the 88 newly vaccinated girls knew the number of doses for the vaccine (two doses) and reported that they were willing to receive their second dose. Among the 57 that did not get vaccinated after expressing willingness, 23/57 were prohibited by their caretakers and 34 were not linked to the health facility for reasons such as traveling out of Kisenyi.

### Significant stories of success or failure in the peer-to-peer education approach for increasing HPV vaccination acceptability

#### Story of success in approach: Adolescent peer educators can successfully influence vaccine knowledge and acceptance among unvaccinated peers

The most significant stories documented during the weekly mentorship meetings with the APEs are summarized below and describe how the APEs influenced unvaccinated peers to accept the HPV vaccine by sharing knowledge about the HPV vaccine and linked interested and willing unvaccinated peers to the community health workers.

*“For me I have so far met four of my friends*, *3 from school and 1 from the community*. *Among the 3 that I met at school*, *two of them* *told me they received the two doses of the HPV vaccine*. *The other one* *told me that she received one dose…*

***Sharing knowledge about the HPV vaccine***

*For the one that I met in the community on Wednesday*, *I went there to pick a book (from her house) and I asked her if she received the HPV vaccine*. *She asked me what the HPV vaccine was*, *and I told her it prevents cervical cancer*. *She told me that I did not know what I was talking about*. *She first asked me how many doses they receive*, *I replied to her “two doses”*. *She then told me that “for the first dose they (health workers) give you the real virus and with the second dose that is when they give you the medicine”*. *I told her that is was not true*! *I told her that I received it a long time ago when I was still in primary six and for her*, *she was in primary five*. *Then*, *I asked her if she is willing to receive the HPV vaccine*, *she said no*. *She said that she cannot receive it because “it is fake”*. *I told her it’s not fake*, *and we can go and talk to the VHT*, *and she gets vaccinated*. *She still said that she does not want*.

*I insisted*.

*I told her that at this age you cannot get cervical cancer*, *but you may get it in future*. *She was shocked and then she told me that she hadn’t reached her future*. *I told her that the future is not far because time is passing by day and night*. *She then said that I had made a good point*, *and I might be right*. *She asked me how one can tell that they have cervical cancer*. *I told her signs are bleeding after having sex with your partner and she was shocked and said she never knew about it*. *I told her the second one is a bad smell (in the private parts)*. *She said that since I have told her all of those (signs) she is going to try and see how she can talk to the VHT*, *and she takes the vaccine*.

***Linking interested and willing unvaccinated peers to the community health workers*.**

*…*.*so*, *she finally accepted and said we shall go to the VHT*. *But she said that she had to first talk to her mum*. *By then her mum was inside the house sleeping and I don’t know whether her mum heard our discussion*. *I am planning to go back today and see if the mother agrees for us to go with the VHT to the health facility”*. (**APE 14 years, mentorship meeting).**

[When the APE went back to the mother, the mother agreed for her daughter to be vaccinated]

#### Story of failure in approach: Adolescent peer educators may not ably overcome all intentional and motivational barriers along the vaccination pathway

The APEs also described some instances when they did not successfully explain the benefits, vaccine safety or failed to interest unvaccinated peers to receive the HPV vaccine or convince the caretakers to allow the girls to be vaccinated.

. *I promised that I would get seven girls*. *Six refused to get vaccinated*. *Two said that they had received their first dose and the other four said that they do not want to get vaccinated because their parents refused*.

**Facilitator:** Tell us how it all happened

***Unsuccessful explanation of vaccine safety***

*For the four that refused to get vaccinated*, *I asked them “why did your parents refuse”*?

*Two of them* *told me that their mother told them that when “they inject you with the HPV vaccine you don’t give birth”*. *I asked them*, *“Can I talk to your mother*?*”*, *then they said that their mother is so tough*, *and I can’t talk to her*.

*I told them I will try my best*. *Then we went to their home*, *and I talked to their mother*, *I explained to her*. *I told her that the vaccine prevents your child from cervical cancer and in future she cannot get that disease*. *Then the mother said “aah for me I am scared”*. *I asked her*, *“why*?*” She told me that while they were still young*, *their friend was vaccinated*, *and she died*. *I told her*, *“Maybe she (her childhood friend) had another disease and that is why she had died”*. *Then the mother said*, *“Let me first think about it”*.

[After a day, the APE met the mother, and she still did not allow her daughters to get vaccinated]

***Failure to interest unvaccinated peers in the HPV vaccine***

*Then the other two*, *I talked to them again on another day and they refused completely*. *I asked them “why don’t you want to go and be vaccinated”*. *They told me that they don’t want that vaccine*, *because they (health workers) already vaccinated the girls and gave them a pink card*. *I told them that is not the HPV vaccine*, *that was Measles-Rubella*. *I told them that Measles-Rubella is different from HPV vaccine*. *They asked me*, *“how is it different”*? *I told them that Measles-Rubella vaccine prevents against Measles and Rubella and HPV vaccine prevents against cervical cancer*. *They still said that they cannot go to receive the HPV vaccine because they already injected them*. *I told them that what they received is not the vaccine I was talking about*. *They still refused*, *I stopped the conversation and bid them farewell*.

**(APE, 14years, 4the mentorship meeting)**

### Reasons for success of the peer-to-peer education approach

Five themes emerged among the reasons why the peer-to-peer education approach was a success; explaining benefits of the vaccine in relation to childbirth, managing expectations of injection pain, commitment of the APEs to follow up and remind unvaccinated peers in the community, linkage of unvaccinated peers to the nearest public health facility and support of the VHTs to engage caretakers.

#### Explaining benefits of the vaccine in relation to childbirth

The APEs noted that most of the unvaccinated peers that had ever heard of the vaccine feared that the HPV vaccine would lead to “failure to give birth in the future”. The peers informed them that cervical cancer affects the reproductive health system and can affect childbirth, but the HPV vaccine prevents cervical cancer and protects their reproductive system, and this convinced several of the unvaccinated peers to get vaccinated.

*“I got one girl; I asked her whether she was vaccinated against cervical cancer… I told her that in future she would not suffer from cervical cancer, she could give birth, and she wouldn’t have any problems when giving birth. She asked me where she could get vaccinated from and I told her that we could go to the city council hospital” (* **APE, 14years, mentorship meeting)**

#### Managing expectations of injection pain

One of the most immediate fears among the unvaccinated peers was the vaccine injection. The APEs noted that while the existing health education message said that the injection does not cause pain, most of the unvaccinated peers were not convinced and had to be told that the injection pain was less of a problem compared to the benefits of the vaccine and this helped the girls to overcome fears of the injection pain. It was noted from the mentorship meeting discussions that it is important to be factual and to keep engaging unvaccinated girls about injection pain fears up to the final point of getting the injection because the unvaccinated peers can change their minds at the last minute and refuse the vaccine.

*“She asked me whether the injection pains. I (APE) told her that it pains a little bit but she will gain more. I asked her whether her father would allow her if I talked to him…..then I went and talked to her father and he accepted for her to be vaccinated”* (**APE, 14years, mentorship meeting)**

*“…*..*then she said that she was willing to be vaccinated*. *She was at her father’s workplace when we went to fetch her to go to the health facility*, *she (unvaccinated peer) first hide from us*! *We went round until we found her…*.*Her father then told us to go with her*. *When we were crossing the road*, *the girl feared and she said that she didn’t want to be given an injection…*.*I had to plead with her to come with me*. *When we reached the city council hospital*, *she first refused the injection…****(APE*, *14 years*, *mentorship meeting)”*.**

#### Commitment of the APEs to follow up and remind unvaccinated peers in the community

During the FGDs with the newly vaccinated peers, the girls said that they had accepted to be vaccinated because “their friends had told them”, and that the APEs would take time to explain everything well and even talk to the caretakers or parents to ask for permission to go with the unvaccinated peers to the health facility.

*“I didn’t want to come but she (APE) told me to come, and they vaccinate me because the injection will help me. She didn’t give me anything, but she is my good friend* ***(*Newly vaccinated peer, FGD4)**

*“Peer educators know how to talk to the children and the children can accept to be vaccinated*. *But the nurses stay in hospitals*, *and they do not come in the community* **(Newly vaccinated peer, FGD 6)**

*“I think the APEs are very helpful in the community because some parents don’t know about any vaccine*, *but those peer educators come and help them to know more about the vaccines and they (APEs) talk to very many people”* **(Newly vaccinated peer, FGD 3)**

#### Linkage of unvaccinated peers to the health facility and support of VHTs to engage caretakers

The APEs highlighted that the availability of the VHTs to support the unvaccinated girls to access the vaccine at the health facility on the dedicated vaccination day was beneficial.

“*The peer educator told me about the vaccination, and she told me that the injection prevents cervical cancer and I accepted. But my mother refused. So, on the day of the vaccination the VHT came home and talked to my mother and my mother permitted me to come (to the health facility)”* **(Newly vaccinated peer, FGD1)**

This was coupled with VHTs being available to engage caretakers in case of caretaker refusal to have their daughters vaccinated since the APEs didn’t have all the skills and power to influence caretakers.

*“I met two girls,…one is our neighbor…we (with another APE) went and talked to her mother, but she said that she didn’t understand anything. When the VHT came, he asked her if we had explained to her. The mother told him (the VHT) that we explained but she didn’t understand anything. Then he (VHT) explained to the mother again and the mother accepted her daughter to be vaccinated. The girl first feared…we told her that it (injection) hurts a little. When Wednesday reached (the day of health facility in-reach), they brought the girl for vaccination. She received the vaccine and she said that it didn’t pain her*” **(APE 12years, mentorship meeting)**

The newly vaccinated girls also described how being escorted to the health center by the APEs enabled them to “safely cross the busy road going to the health center” and this further exemplified the role of health system linkage and access to the vaccines.

### Reasons contributing to the failures of the peer-to-peer education approach

Four themes emerged as reasons why the APEs did not convince some of the unvaccinated peers to receive the HPV vaccine; 1) caretakers are the final decision makers in the vaccination pathway, 2) confusion of the HPV vaccine with the tetanus or measles-rubella vaccine, 3) limited physical access to the vaccine in a health facility-based model, and 4) limited repeat socializations between APEs and out-of-school peers.

#### Caretakers are the final decision makers in the vaccination pathway

The APEs highlighted several instances where caretakers prohibited vaccination even when their daughters were interested and willing to be vaccinated and sometimes caretakers changed their minds on the day of vaccination. Some caretakers also didn’t want the APEs to engage with their children because they thought that the APEs could “negatively influence” their children. Quantitative data from the newly vaccinated peers showed that 40/88 (45.6%) of the newly vaccinated peers were influenced by the APE with follow up made by the APE to the caretaker to seek permission. The APEs overcame the caretaker hesitancy by explaining to them the safety of the vaccine. However, explaining vaccine safety comprehensively to a caretaker was often challenging to the APEs and the VHTs had to intervene.

*“…she is 12years and then I asked her if she got the HPV vaccine? She asked me, what is HPV vaccine? I told her that it is the vaccine which prevents cervical cancer…she told me that “I would like to come but I don’t know whether my mother will accept” …”* **(APE, 12 years, mentorship meeting)**

*“…at that time their father came*, *he said that we (APEs) are the ones that make their children fall sick by vaccinating them*. *He chased me away*. *I told him that he got me wrong and asked for a chance to explain to him*. *But the father told me to leave him alone saying that he is older than me therefore there is no way I can explain to him*.*”* **(APE, 13 years, mentorship meeting)**

*“*…*I went with the VHT to the girls’ parent’s home on the day of vaccination*. *When we reached there*, *the VHT explained to the mother (that we would escort the daughter to the health center)*. *The mother said that she doesn’t want her girls to be vaccinated because the vaccines are fake*. *The VHT explained to the mother everything*, *and the mother accepted*. *On Thursday*, *I went back fetched the girls and we went to city council hospital and the girls got their first dose of the HPV vaccine*. *When we returned to their home*, *the mother who had initially accepted the girls to get vaccinated changed her mind and she questioned the girls why they had got vaccinated…*. *It seems the neighbours influenced the mother to change her mind”* **(APE, 12 years, mentorship meeting)**

*“Some parents don’t want their children to move out or to talk to people they don’t know*. *So*, *the APE might come wanting to talk to the girl and the parent chases the APE away”* **(newly vaccinated peer, FGD2)**

#### Confusion of the HPV vaccine with the tetanus or measles-rubella vaccine

Sometimes the unvaccinated peers and the caretakers confused the tetanus vaccine and measles-rubella vaccine for the HPV vaccine. While some girls thought they had received the HPV vaccine, upon detailed engagement with the APEs, it was learnt that other vaccines were received but not HPV vaccine and this could easily be differentiated based on the vaccination cards that the peers had, with purple for HPV vaccine and pink for tetanus vaccine. Prior to our intervention, a national measles-rubella (MR) vaccination campaign had been implemented among 2-15year olds in communities and schools and most girls thought that the MR vaccine was the same as the HPV vaccine.

*“….when we were crossing the road, the girl said that she doesn’t want to be given an injection because she was already vaccinated. I asked her the color of the card she had, and she said it was pink for (measles-rubella). She said that she had a pink one and a yellow one. I told her that I was talking about the purple one and she asked me what the difference was…*” **(APE, 13 years mentorship meeting)**

*“We were playing then I asked them*, *have you ever heard about the HPV vaccine*? *One of them asked me*, *“what do you mean”*? *I told her that…it prevents cervical cancer*. *Then she told me*, *that she was vaccinated*, *and she got a card*. *But she was telling me about the pink card for measles-rubella)…*.*Then I told the girl that I meant the purple card*. *I showed the girl my purple card and she said to me “no*, *I do not have this one…”*. *I went and talked to her parents*. *Her parents told me I could take her for HPV vaccination”* (**APE, 11 years, mentorship meeting)**

#### Limited physical access to the HPV vaccine in a health facility-based model

When the HPV vaccine is only offered at the health facility and in school campaigns, there is limited access. The APEs described how the girls that do not go to school usually work in restaurants and as house maids and could not leave their work to go to the health facility and line up for a vaccine. Also related to physical access to the health facility was the uncertainty of the girls’ safety on their way to the health facility because the road to the health facility usually has many cars and motorcycles and the girls did not feel safe crossing the road on their own. Some feared that they could get kidnapped on their way to the health facility since their slum community had high crime rates while others said that the health facility was far.

*“…my friend refused to get vaccinated. She said she was busy, and that the health facility is very far, and she cannot walk there”* **(APE, 11 years, mentorship meetings)**

*“The health facility is not far*, *only that the vehicles are so many”* **(Newly vaccinated peer, FGD6)**

*“…we first went to fetch Agnes so that we take her to the city council health facility*. *But then*, *Agnes’ mother said that Agnes had received the vaccine*, *yet Agnes told us that she had not been vaccinated against HPV…*.*The mother told us to leave her children alone because they were still doing house chores”*. **(APE, 15 years mentorship meeting)**

#### Limited repeat socialization between APEs and out-of-school peers

All the APEs were in-school and had limited opportunities to engage with their out-of- school peers who were working in restaurants or as house maids hence had limited opportunities to socialize and build friendship. In addition, some girls only come in the community for school and during the school break they travel to their upcountry homes which still limits repeat socialization among peers in the community.

*“… .we (two APEs) talked to the girls that work in the restaurant and they had accepted (to be vaccinated). On the day of vaccination, we went to fetch them from the restaurant at 3pm (to be taken for vaccination at the health facility). They were three girls, but on our way, two of them escaped from us and we still do not know why. We have not gone back to them because we fear talking to them again since we do not know them much* **(APE, 13years, mentorship meeting).**

*“For me the challenge that I see we are facing is that we anticipated having many girls during the Christmas holiday break*, *but we later found out that many girls were sent to villages for holidays*. *They go to school here in the city but they go for their holidays in the village so that is why we have got few numbers this December*. **(APE, 12 years, mentorship meeting)**

## Discussion

This study piloted an intervention to improve HPV vaccine acceptance through peer-to-peer education among adolescent girls in the urban poor settings of Kisenyi, Kampala, Uganda. APEs were able to engage and communicate with a high acceptance and uptake of the HPV vaccination which demonstrated the potential of the peer-to-peer education and support to expand the reach of the HPV vaccine in an urban poor community. These findings highlight the opportunity of social influence [44] and social connectedness among vaccinated friends and neighbours as informed sources of knowledge about cervical cancer and the HPV vaccine as well as referral-a recommended cue to action [11, 30]. Positive social influence, friendship, social connections and repeat socialization especially among school going adolescents enabled the success of the peer-to-peer education approach.

APEs were able to communicate the benefits of the HPV vaccine using health education messages which were co-created with the APEs and VHTs and appropriately packaged to address their needs as well as the gaps in existing messages including the benefits and anticipated risks (injection pain) and fears such as failure to have children [45, 46]. Many unvaccinated girls had concerns about the HPV vaccine leading to infertility which can be a major barrier in a community where fertility is highly valued. Appreciating these fears and addressing them in the messaging was critical in gaining acceptance and emphasizing the importance of understanding of perceived benefits, perceived barriers and cue to action in promoting the HPV vaccine [40]. All the newly vaccinated peers knew the correct number of HPV vaccine doses and were willing to receive their second dose, which demonstrates that the APEs were indeed an informed and influential source of knowledge.

The APEs had a good grasp of the message because they were involved in co-creating the message, which was structured and availed to them for reference. They were also trained and attended mentorship meetings to share their experiences and challenges and explore solutions to strengthen their confidence and ability to engage with their unvaccinated peers and families. Through the mentorship meetings the intervention team was also able to listen, learn and make adaptations to ensure responsiveness to the challenges faced by the APEs while executing their work and to encourage the APEs in their tasks. This continuous learning, group participation and sharing also enabled documentation of process and refinements of the peer-to-peer engagement process for optimization as applied in the human centered design [39].

For peer-delivered interventions, the selection process is important in ensuring that appropriate individuals who are well connected and acceptable to the communities are identified. The APEs in this intervention were selected in close consultation with the VHTs who had a good grasp of the sociocultural setting and knew the adolescents. This ensured that the APEs selected had the right attributes such as being committed and open to taking on leadership responsibilities-qualities that have been shown to be important for peer educators and expert clients [47, 48].

In this study we noted that caretakers were the final decision makers for HPV vaccination uptake among their daughters as has previously been shown in other studies [49–51]. Caretakers and parents usually have fears that their children will not give birth when they receive the vaccine [52]. In our study, some unvaccinated peers indicated that they were willing to take up the vaccine, but the caretaker(s) refused to allow them. The intervention of the APEs and the backup support of the VHTs in engaging the parents and caretakers emphasizes the need for more active engagement of communities with targeted family support in increasing HPV vaccine uptake. The peer-to-peer model can be used as a mechanism to identify caretakers that are hesitant and that need additional engagement and education.

While the Uganda Immunization Act provides for compulsory immunization of children, women of reproductive age and other target groups against immunizable diseases [53], immunization of adolescent girls against HPV without parental consent has not been fully operationalized. From literature, caretakers are not only parent e.g., grandmothers may influence the decision and could deter girls from vaccinating when they have doubts about the vaccine [54]. It is thus necessary to ensure that all key influencers are engaged in order to overcome the multiple layers of hesitancy [55]. In recognition of the multiple stakeholders within the vaccination efforts, in December 2018, the Uganda Ministry of Health launched a national strategy to overcome hesitancy and improve HPV uptake through a multi-sectoral collaboration-a strategy that has increased HPV vaccine acceptance and uptake in the school-based campaigns [8].

One of the most common reasons for not vaccinating was that the girls did not know about the HPV vaccine, and some had not been offered it. This was an indication of gaps in community mobilization, education, and access to all the girls in the schools and in the community. This further compounds the problem of vaccine hesitancy and affects the pathway from motivation to vaccination as depicted in the WHO increasing vaccination model [24]. In our study we noted that some girls who were willing to be vaccinated could not be linked to the health facility because they travelled away or could not access the health facility as has been recommended in the journey to health and immunization framework [56]. Such missed opportunities have been highlighted in urban poor settings which have highly mobile and transactional populations. This further highlights the importance of health system factors in enabling vaccination uptake [57].

The peer-to-peer education process involved following up girls that expressed interest to get vaccinated to ensure they were linked to the health facility and supporting them until they received all the doses of the vaccine because the girls or caretakers could change their minds along the decision-making pathway. With in 12-weeks of follow up, 88 girls received their first dose of the HPV vaccine which shows the potential of the growing network-based peer advocacy [34]. This could be further expanded and sustained as more girls complete their vaccination and can join the network of peer educators for HPV vaccine promotion. Studies have shown that once vaccination is started, there is increasing likelihood of completion and taking up other beneficial vaccines, so this intervention could have spillover effects in increasing uptake of other vaccinations along the life course. There is also growing evidence that a single dose series is beneficial, and this may further enhance HPV vaccination uptake in the future [58].

To optimize the peer-to-peer education approach in HPV vaccine uptake, there are several contextual factors that need to be considered. For example, the need to be aware of the right APE attributes like commitment and the environmental support like availability of VHTs to link APEs to the health system as well as engagement of the service providers to ensure preparedness to serve the referred girls. In this intervention, engagement with the health center to ensure a dedicated vaccination day and supplies was a critical factor in the success of the intervention. Though not the primary focus of our study, this intervention reveled various dynamics of a “girl child” in this urban slum setting of Kisenyi and the extent to which the APEs had the space to fully apply their leadership and advocacy abilities in a community that sometimes viewed the APEs as children.

During dissemination meetings, stakeholders proposed that organizing the APEs in “an HPV vaccine promotion club” in each school or “HPV vaccine leaders” to work in the community with linkage to the local health facility to reach out-of-school girls could help to sustain the gains of the approach. Such contextual and implementation factors can guide the design and application of the approach in different contexts maximize the functionality of the peer educators and increase the effectiveness of the approach to contribute to the broader adolescent sexual and reproductive health and the life-course immunization agenda [16, 59].

This study adds value to the learning on how we can leverage the voices, energy and creativity of adolescents and youth to improve HPV vaccine acceptance and uptake and other services for young people. Other studies have shown the benefit of community-led approaches such as peer educators in the scale up of public health interventions [31]. This positive recognition of the role of adolescent peer educators was described by the newly vaccinated peers who indicated that the peer educators were valued since they were friendly, they worked in the community, talked well with fellow girls, and could be trusted.

### Considerations for adoption

As part of the adolescent peer educator support system, we held weekly mentorship meetings with the APEs and involved the VHTs in all the study processes as a community support structure. Working with the VHTs helped embed the approach in community structures, ensure its responsiveness and could enhance its sustainability. However, the mentorship model used in our study was expensive in terms of time input on weekends by the Researchers and VHTs, providing transport refund to the VHTs and facilitating the weekly mentorship meetings with the APEs. The duration of this intervention may have been too short to fully assess the changing support needs for the APEs—we anticipate that less frequent and shorter meetings could have worked overtime as the APEs improved their grasp of the interventions. More work is needed to explore potential adaptations to this model and to assess its sustainability and broader impact.

To ensure equitable representation of APEs from the different geographical zones of the slum, we engaged the VHTs to identify adolescents who had completed the HPV vaccine doses through a community mapping exercise. However, in our study, some minority ethnic populations in Kisenyi slum could not have representation among the trained APEs since no adolescents from these “minority ethnic populations” had completed the HPV vaccine doses in the first place and this presented an equity issue.

We recognize the set back that the COVID19 pandemic had on immunization programs globally including the Uganda HPV vaccination -where school-based delivery of the vaccine was halted due to the COVID19 restrictions. On March 30, 2020, the Ugandan government issued restrictions on movement that lasted until May 22, 2020. During that time, newly vaccinated girls that were eligible for their second dose could not access the vaccine until movement restrictions were lifted.

### Study strengths and limitations

This was an exploratory study to test the feasibility of the peer-to-peer education approach. From the qualitative approach we derived a thick description of processes to identify intervention elements that are promising for success and elements that are a challenge. We could not determine the extent to which vaccination uptake increased due to the peer-to-peer education approach due to the lack of a counterfactual. The APEs were identified through the initial mapping exercise led by the VHTs and this could have introduced selection bias towards those who were known to them or in the more familiar settings. This could have limited the representativeness of the adolescents from this urban poor community and reduced the depth of experiences learned from our sample. The decision-making process of the caretakers was also not explored since no caretakers were interviewed in this study. We attempted to use one set of “health message content” for the APEs to use while engaging unvaccinated peers in the community. However, we were not able to evaluate this against the existing health message content and the extent to which each element in the message could lead to similarities or differences in results. In addition, the health message was co-created by the intervention team and APEs hence the thought process and ideas of the APEs could have been influenced by intervention team. During the co-creation and training processes, APEs were encouraged to share their ideas first before the intervention team proposals to minimize intervention team influence. These gaps could be addressed through additional research to refine the intervention and assess its impact in a larger study with more controlled experimental designs and comparison group to inform generalizability of findings and explore applicability in diverse settings.

## Conclusion

Peer-to-peer education is a feasible approach to promote HPV vaccine acceptance among adolescents and their caretakers and can potentially increase HPV vaccine uptake. Unvaccinated girls were identified, engaged, convinced, and supported to overcome vaccine hesitancy. During demand creation for the HPV vaccine all key influencers of the vaccination decision making pathway should be engaged especially the caretakers to overcome hesitancy at multiple levels. Linkage to the health system through the VHTs and a health facility ensured vaccine physical access for the girls that needed it. There is need to assess the efficacy of peer-to-peer education approach in larger studies, explore how to sustain the approach and grow the network of peer educators in routine practice.

## Supporting information

S1 Checklist COREQ (COnsolidated criteria for REporting Qualitative research) checklist. (PDF)

S1 Text Needs finding discussion guide for adolescent girls aged 10–15 years. (DOCX)

S2 Text Co-creation of the peer-to-peer education intervention and training process of the adolescent peer educators (APEs). (DOCX)

S3 Text Focus group discussion guide for newly vaccinated adolescent girls aged 10-15years. (DOCX)

S1 Table Table with an expanded list of diverse quotes from the data analysis. (DOCX)
