# Supplementary material for: Improving HPV vaccine acceptance through peer-to-peer education among adolescent girls in the urban poor settings of Kisenyi, Kampala, Uganda
Source: PLOS Glob Public Health. 2024 Dec 5;4(12):e0004007. doi: 10.1371/journal.pgph.0004007 (PMC11620462; doi:10.1371/journal.pgph.0004007)
Supplement: S1 Text — (DOCX) [file pgph.0004007.s002.docx]

# **S1 Text**

# **Needs finding discussion guide for adolescent Girls Aged 10-15years**

| Date of the interview: |  |
| --- | --- |
| Name of interviewer: |  |
| Venue of the interview: |  |
| Name of the community |  |
| Category of respondents |  |

Note: The targeted adolescents are those that in their respective categories will discuss their perceived susceptibility and severity of cervical cancer, perceived benefits of HPV vaccine, perceived barriers to HPV uptake, cue to action and self-efficacy for HPV uptake.

1. Have you heard of cervical cancer?
2. How did you hear /learn to know about it?
3. How does a woman get cervical cancer?
4. Who is at risk of suffering from cervical cancer?
5. How does one know that they have cervical cancer? Probe for age most affected, signs and symptoms.
6. Is cervical cancer a serious disease? Explain
7. Do you think cervical cancer causes death? Probe for any persons they know who have died of cervical cancer.
8. How can cervical cancer be prevented?
9. Have you had of the HPV vaccine?
10. Where did you get the information from
11. What was told to you? (*Interviewer to pay attention to any misinformation, myths and misconceptions*)
12. Do you think HPV is beneficial?
13. I know that you received the two dozes of the HPV vaccine, explain to me why
14. Where were you vaccinated from
15. Do you believe the HPV vaccine prevents cervical cancer?
16. Should every young adolescent receive the HPV Vaccine
17. We have heard that some adolescent girls resist/ refuse or delay to take up the HPV vaccine. What is your experience with that in this community?
18. Why do adolescent girls resist/refuse or delay to take up the HPV vaccine
19. Is there any resistance against HPV vaccination from the Parents? Please explain
20. Are there girls out of school in this community? Probe for age and reasons why
21. What factors enabled you to take up the vaccine

*Probe for role of health education*

*Probe for role of caretaker/ guardian*

*Probe for role of peers*

*Probe for accessibility of the vaccine (physically and affordability)*

*Probe for where they received the vaccine like health facility or outreach. Was it considered near?*

*Probe for encouragement to go for the HPV vaccine by anyone*

1. Who conducts the HPV vaccination in this community?
2. Suggestions ways that you think can help increase the Uptake of HPV vaccination services among adolescents in this community

Thank you
