# Supplementary material for: Improving HPV vaccine acceptance through peer-to-peer education among adolescent girls in the urban poor settings of Kisenyi, Kampala, Uganda
Source: PLOS Glob Public Health. 2024 Dec 5;4(12):e0004007. doi: 10.1371/journal.pgph.0004007 (PMC11620462; doi:10.1371/journal.pgph.0004007)
Supplement: S2 Text — (DOCX) [file pgph.0004007.s003.docx]

**S2 Text**

**Co-creation of the peer-to-peer education intervention and training process of the adolescent peer educators (APEs)**

The 18 girls, ages of 10-15 years, who had previously received two (complete) doses of the HPV vaccine worked with the intervention team to co-create a structured health education message (the message) using the human centered design approach. The intervention team first presented the discussion findings and co-created with the APEs. The findings showed that the main enablers of vaccination were receiving parent’s advice, receiving friend’s advice and the vaccine being available or being brought to school. Barriers to HPV vaccination included misinformation that the vaccine causes infertility and lack of information about the benefits of the vaccine.

After sharing the findings from the needs finding discussion, the HPV health education message co-creation process followed. This was informed by the constructs of the Health Belief Model namely, perceived susceptibility, perceived severity, perceived benefits, perceived barriers, cues to action and self-efficacy and the wording of the messaging was informed by the findings from the needs finding discussion. The co-creation process aimed at developing a message that communicates the risks of HPV, the benefits of the HPV vaccine, that the APE has previously received the vaccine and it is safe and how the vaccine can be accessed. After co-creation of the message, the APEs were trained over two days by the intervention team on how to identify girls aged 10-13years, confidently inquire about their peers’ HPV vaccination status and number of doses received and deliver a structured health education message to unvaccinated peers. The APEs were provided with tips on how to deliver the message in a structured way, coherently and chronologically.

Through brainstorming sessions in the training, the APEs suggested three approaches to reaching unvaccinated peers i.e., through 1) home visitation, 2) play time engagement and 3) holding conversations at the water point where many girls gather to fetch water for household use. The message co-creation and APE training lasted for four weeks (October 8^th^-November 8^th^, 2019). The trained APEs were commissioned through a “leadership commitment” ceremony hosted by the community youth leader. Each APE was asked by the intervention team to identify as many unvaccinated peers in their community as possible. Through engagements, the APEs and intervention team set a target for each girl to influence at least 10 unvaccinated peers for HPV vaccination over a 12-weeks period.

**The structured health education message**

Peer educators and facilitators were actively involved in preparing the health education message. The final health education message was:

1. There is no cure for cervical cancer

2. HPV-vaccine prevents cervical cancer

3. Advise parents to vaccinate their children

4. To avoid cervical cancer, one should avoid early sex and be vaccinated

5. Cervical cancer leads to bad smell in the private parts

6. Prevention is better than cure

7. Parents should be told the benefits of the PHV-vaccine

8. The HPV-vaccine is safe and not very painful when they inject you

9. The time I give birth I will be free from cervical cancer if I am vaccinated early (10-13 years)

10. The HPV vaccine is free at Kisenyi Health Centre IV

11. Are you willing to take up the vaccine?

12. Let me take you to my VHT
