# Supplementary material for: Improving HPV vaccine acceptance through peer-to-peer education among adolescent girls in the urban poor settings of Kisenyi, Kampala, Uganda
Source: PLOS Glob Public Health. 2024 Dec 5;4(12):e0004007. doi: 10.1371/journal.pgph.0004007 (PMC11620462; doi:10.1371/journal.pgph.0004007)
Supplement: S3 Text — (DOCX) [file pgph.0004007.s004.docx]

# S3 Text

# Focus group discussion guide for newly vaccinated adolescent girls aged 10-15years

| Date of the interview: |  |
| --- | --- |
| Name of interviewer: |  |
| Venue of the interview: |  |
| Name of the community |  |
| Category of respondents |  |

Note: The targeted adolescents are those that in their respective categories will discuss their perception towards the design of the peer education and peer group participation solution that influences their vaccine uptake. Perceptions towards the peer education message, role of peer educators and other processes will be enlisted.

1. Tell us how you made the decision to take up the first doze of the HPV vaccine in this community.

*Probe: Why did you get vaccinated?*

*Probe: Who encourage you*

*Probe: Where did you receive the vaccine from?*

*Probe: How many doses have you received (if one, ask about date for the next dose)*

1. Explain the role of the peer educators in your decision to take up the HPV vaccine?

*Probe: What are the benefits of peer educators?*

*Probe: What us the name of the peer educator that referred you for vaccination?*

*Probe: What did the peer educator tell you?*

*Probe: What are the challenges of working with peer educators?*

1. What is your perception towards peer-to-peer education as a solution to increase HPV uptake?

*Probe: Can it work in this community?*

*Probe: Can it be accepted in this community?*

1. What design features of peer-to-peer education do you like?

*Probe: How the peer groups are arranged*

*Probe: How the peer groups work in the community*

*Probe: The best characteristics of a peer educator*

Thank you.
