# Supplementary material for: Improving HPV vaccine acceptance through peer-to-peer education among adolescent girls in the urban poor settings of Kisenyi, Kampala, Uganda
Source: PLOS Glob Public Health. 2024 Dec 5;4(12):e0004007. doi: 10.1371/journal.pgph.0004007 (PMC11620462; doi:10.1371/journal.pgph.0004007)
Supplement: S1 Table — (DOCX) [file pgph.0004007.s005.docx]

**S5 Table**

**Table with an expanded list of diverse quotes from the data analysis**

|  | **Reasons for success of the peer-to-peer education approach.** |
| --- | --- |
|  | **HPV vaccine prevents cervical cancer which affects the reproductive system of women and girls.** |
| Q1 | *“I got one girl; I asked her whether she was vaccinated against cervical cancer... I told her that in future she would not suffer from cervical cancer, she could give birth and she wouldn’t have any problems when giving birth. She asked me where she could get vaccinated from and I told her that we could go to the city council hospital” (***APE, 14years, mentorship meeting)** |
| Q2 | *“...she (APE) told me that she is going to take me to get vaccinated against cervical cancer so that I don’t get the disease (cervical cancer). She (APE) told me that I will be able to give birth and I will not get the disease. Then I told the APE that we will go for vaccination…”* **(Newly vaccinated peer, FGD3)** |
| Q3 | *“I told the girls (friends of the APE that are not vaccinated) that if they get vaccinated against cervical cancer, in future they would be able to give birth. I told them that the virus may come in the future if they are not vaccinated….The girls understood that it (cervical cancer) is a serious disease and they agreed to get vaccinated”* *(***APE, 13years, mentorship meeting)**. |
| Q4 | *“We were at school, and I asked one of our neighbors whom I am in the same class whether she was vaccinated against HPV. She told me that she has never heard of it and that she was not interested….Then, I told her the benefits of the HPV vaccine and she said she was willing to get vaccinated. I then told her that I am going to talk to VHT who will take her to the health facility for vaccination” (***APE, 13years, mentorship meeting**) |
|  | **Managing expectations of injection pain** |
| Q1 | *“She asked me whether the injection pains. I (APE) told her that it pains a little bit but she will gain more. I asked her whether her father would allow her if I talked to him…then I went and talked to her father and he accepted for her to be vaccinated”* (**APE, 14years, mentorship meeting)** |
| Q2 | *“...then she said that she was willing to be vaccinated. She was at her father’s workplace when we went to fetch her to go to the health facility, she (unvaccinated peer) first hide from us! We went round until we found her….Her father then told us to go with her. When we were crossing the road, the girl feared and she said that she didn’t want to be given an injection….I had to plead with her to come with me. When we reached the city council hospital, she first refused the injection…”* **(APE, 14 years, mentorship meeting)** |
| Q3 | *“…when I went to the girl’s home (to take her for vaccination) she changed her mind. Her mother was at home at the time, and she (the mother) told the girl to get dressed and go with us to the health facility. Then the girl said that she was vaccinated and that she even has the (HPV vaccine) card….I think the truth is that she feared the injection”.* **(APE, 13years, mentorship meeting)** |
| Q4 | *“For me I didn’t accept at first. But as days passed, she (APE) continued telling me to go with her and I get vaccinated at the city council health center and the injection will not pain. Then I finally accepted and went with her (the APE)”.* **(Newly vaccinated peer, FGD2)** |
|  | **Commitment of the APEs to follow up with their unvaccinated peers in the community.** |
| Q1 | *“I didn’t want to come but she (APE) told me to come, and they vaccinate me because the injection will help me. She didn’t give me anything, but she is my good friend* ***(*Newly vaccinated peer, FGD4)** |
| Q2 | *“Peer educators know how to talk to the children and the children can accept to be vaccinated. But the nurses stay in hospitals, and they do not come in the community* **(Newly vaccinated peer, FGD 6)** |
| Q3 | *“I think the APEs are very helpful in the community because some parents don’t know about any vaccine, but those peer educators come and help them to know more about the vaccines and they (APEs) talk to very many people”* **(Newly vaccinated peer, FGD 3)** |
| Q4 | *“A fellow peer can beseech you (unvaccinated peer) but an adult health worker thinks that they cannot beg a young girl (***Newly vaccinated peer, FGD1***)* |
|  | **Linkage of unvaccinated peers to the health facility on the vaccination day** |
| Q1 | “*The peer educator told me about the vaccination, and she told me that the injection prevents cervical cancer and I accepted. But my mother refused. So, on the day of the vaccination the VHT came home and talked to my mother and my mother permitted me to come (to the health facility)”* **(Newly vaccinated peer, FGD1)** |
| Q2 | *“I met two girls,…one is our neighbor…we (with another APE) went and talked to her mother, but she said that she didn’t understand anything. When the VHT came, he asked her if we had explained to her. The mother told him (the VHT) that we explained but she didn’t understand anything. Then he (VHT) explained to the mother again and the mother accepted her daughter to be vaccinated. The girl first feared…we told her that it (injection) hurts a little. When Wednesday reached (the day of health facility in-reach), they brought the girl for vaccination. She received the vaccine and she said that it didn’t pain her*” **(APE 12years, mentorship meeting)** |
| Q3 | *“So, I had gone to the village for holidays and when I came back the APE told me that the VHT had earlier come to my home to take me for vaccination and found when I wasn’t around. So yesterday I went and got vaccinated”* **(Newly vaccinated peer, FGD2)** |
| Q4 | *“…they say that actions speak louder than words. So, when I had what she (APE) had taken others (to get vaccinated), I also accepted”* **(Newly vaccinated peer, FGD4)** |
|  | **Reasons contributing to the failures of the peer-to-peer education approach.** |
|  | **Caretakers are the final decision makers in the vaccination pathway.** |
| Q1 | *“…she is 12years and then I asked her if she got the HPV vaccine? She asked me, what is HPV vaccine? I told her that it is the vaccine which prevents cervical cancer…she told me that “I would like to come but I don’t know whether my mother will accept” …”* **(APE, 12 years, mentorship meeting)** |
| Q2 | *“…at that time their father came, he said that we (APEs) are the ones that make their children fall sick by vaccinating them. He chased me away. I told him that he got me wrong and asked for a chance to explain to him. But the father told me to leave him alone saying that he is older than me therefore there is no way I can explain to him.”* **(APE, 13 years, mentorship meeting)** |
| Q3 | *“…I talked to her (the mother) I told her that can you please accept your child to go and get vaccinated. She (the mother) asked if the injection can affect her child. I said no because it prevents cervical cancer. She asked me, “what is cervical cancer”? Then I explained to her in vernacular. She (the mother) told me that “okay, let me first talk to her dad and when he says yes I will let our daughter go and get vaccinated””.* **(APE, 14 years, mentorship meeting)** |
| Q4 | *“...I went with the VHT to the girls’ parent’s home on the day of vaccination. When we reached there, the VHT explained to the mother (that we would escort the daughter to the health center). The mother said that she doesn’t want her girls to be vaccinated because the vaccines are fake. The VHT explained to the mother everything, and the mother accepted. On Thursday, I went back fetched the girls and we went to city council hospital and the girls got their first dose of the HPV vaccine. When we returned to their home, the mother who had initially accepted the girls to get vaccinated changed her mind and she questioned the girls why they had got vaccinated…. It seems the neighbours influenced the mother to change her mind”* **(APE, 12 years, mentorship meeting)** |
| Q5 | *“Some parents don’t want their children to move out or to talk to people they don’t know. So, the APE might come wanting to talk to the girl and the parent chases the APE away”* **(newly vaccinated peer, FGD2)** |
|  | **Confusion of the HPV vaccine with the tetanus or measles-rubella vaccine** |
| Q1 | *“….when we were crossing the road, the girl said that she doesn’t want to be given an injection because she was already vaccinated. I asked her the color of the card she had, and she said it was pink (for measles-rubella). She said that she had a pink one and a yellow one. I told her that I was talking about the purple one and she asked me what the difference was…*” **(APE, 13 years mentorship meeting)** |
| Q2 | *“We were playing then I asked them, have you ever heard about the HPV vaccine? One of them asked me, “what do you mean”? I told her that…it prevents cervical cancer. Then she told me, that she was vaccinated, and she got a card. But she was telling me about the pink card (for measles-rubella)….Then I told the girl that I meant the purple card. I showed the girl my purple card and she said to me “no, I do not have this one…”. I went and talked to her parents. Her parents told me I could take her for HPV vaccination”* (**APE, 11 years, mentorship meeting)** |
| Q3 | *“She (the mother) told me that they (her two daughters) were not vaccinated and that she has a girl aged 8 years and another one 10 years. She told me that I may take the one aged 10 years for vaccination. Then she said she was still not sure (about the HPV vaccine) because other people (in the neighbourhood) had said that among the children vaccinated against measles-rubella, 100 of them had fallen sick. So, she (the mother) was not sure if she should take her children for other vaccination until she knew the truth. I realized the mother was teasing me and so I left her since she couldn’t let her child get vaccinated”.* (**APE, 14 years, mentorship meeting)** |
| Q4 | *“I then found other three girls and they told me that they were vaccinated. They showed me their pink cards, but I told them that the cards were for the measles-rubella vaccine, but they refused (to get the HPV vaccine). I then left them”* (**APE, 13 years, mentorship meeting)** |
|  | **Limited physical access to the HPV vaccine when only offered at the health facility and in school campaigns.** |
| Q1 | *“...my friend refused to get vaccinated. She said she was busy, and that the health facility is very far, and she cannot walk there”* **(APE, 11 years, mentorship meetings)** |
| Q2 | *“The health facility is not far, only that the vehicles are so many”* **(Newly vaccinated peer, FGD6)** |
| Q3 | *“….we first went to fetch Agnes so that we take her to the city council health facility. But then, Agnes’ mother said that Agnes had received the vaccine, yet Agnes told us that she had not been vaccinated against HPV….The mother told us to leave her children alone because they were still doing house chores”.* **(APE, 15 years mentorship meeting)** |
| Q4 | *“…then she told me that if I wanted to go with her (to the health facility for HPV vaccination) I would first tell her mother….Then I told her that I would meet her mother. Then I talked to her mother but by the time of the vaccination day she couldn’t come with me because she was alone at home and she could not leave the home (unattended to)”* **(APE, 15 years mentorship meeting)** |
|  | Limited repeat socialization between APEs and out-of-school peers |
| Q1 | *“....we (two APEs) talked to the girls that work in the restaurant and they had accepted (to be vaccinated). On the day of vaccination, we went to fetch them from the restaurant at 3pm (to be taken for vaccination at the health facility). They were three girls, but on our way, two of them escaped from us and we still do not know why. We have not gone back to them because we fear talking to them again since we do not know them much* **(APE, 13years, mentorship meeting).** |
| Q2 | *“For me the challenge that I see we are facing is that we anticipated having many girls during the Christmas holiday break, but we later found out that many girls were sent to villages for holidays. They go to school here in the city but they go for their holidays in the village so that is why we have got few numbers this December.* **(APE, 12 years, mentorship meeting)** |
| Q3 | *“Some parents don’t want their children to move out or to talk to people they don’t know. So she (the APE) might come wanting to talk to the girl (unvaccinated peer) and the parent chases the APE away”* **(Newly vaccinated peer, FGD2)** |
| Q4 | “One refused and another said that she will not go for vaccination because she is still busy and she has to fetch water and then wash utensils….She said she was busy and that the vaccination place was very far and she couldn’t go there. She told us to leave her alone and we have not managed to see her again because she lives far away from us”. **(APE, 14 years, mentorship meeting)** |
